# Coordinates-based meta-analysis for vestibular migraine and the underlying mechanisms behind it

**DOI:** 10.3389/fneur.2025.1495423

**Published:** 2025-04-09

**Authors:** Xiaoping Fan, Liang Dong, Hui Li, Kuiyun Wang, Jiying Zhou

**Affiliations:** ^1^Phase I Clinical Research Center, The First Affiliated Hospital of Chongqing Medical University, Chongqing, China; ^2^Department of Neurology, The First Affiliated Hospital of Chongqing Medical University, Chongqing, China; ^3^Department of Neurology, Jintang First People's Hospital, West China Hospital, Sichuan University, Chengdu, China

**Keywords:** coordinate-based meta-analysis, gray matter, vestibular migraine, voxel-based morphometry, insula

## Abstract

**Background:**

Vestibular migraine (VM) is a leading cause of recurrent vertigo episodes. Voxel-based morphometry (VBM) is a reliable technique to analyze structural changes, particularly in gray matter (GM) volume, across various neurological conditions. Despite the growing amount of neuroimaging data in recent decades, a comprehensive review of GM alterations in VM remains lacking.

**Methods:**

We conducted a systematic review of three English-language databases (PubMed, Embase, and Web of Science) and two Chinese-language databases (China National Knowledge Infrastructure and Wanfang) to evaluate existing neuroimaging data on GM volume in VM patients. A coordinate-based meta-analysis (CBMA) was performed using the latest algorithm, seed-based d mapping with permutation of subject images (SDM-PSI), to identify brain alterations across individual studies.

**Results:**

Five studies (103 VM patients, 107 HCs) were included. The CBMA demonstrated a significant reduction in GM volume in VM patients compared to HCs, with peak convergence in the left rolandic operculum (SDM-Z = −3.68, *p*-corrected = 0.004, voxels = 629; Brodmann area 48), extending to the posterior insula. Heterogeneity across studies was low (I^2^ = 19.35%), and no publication bias was detected (Egger’s test: *p* = 0.826).

**Conclusion:**

This meta-analysis confirms reliable GM volume alterations in the posterior insula–operculum region of VM patients. Longitudinal studies with standardized imaging protocols are needed to clarify whether these changes are causes or consequences of VM.

**Systematic review registration:**

https://www.crd.york.ac.uk/prospero/, identifier CRD42021277684.

## Introduction

Vestibular migraine (VM), which is a common neurological disorder characterized by recurrent episodes of vestibular and migraine-related symptoms, is one of the most common causes of vertigo with a prevalence of 1–2.7% among the adult population (1, 2). Despite established diagnostic criteria for VM from the International Headache Society (IHS) and the Barany Society (3, 4), the pathogenesis of VM is not fully understood. The existing hypotheses are derived from migraine pathophysiology (5). There are anatomical connections between the nociceptive and vestibular systems at the brainstem and cortical level (6–9). Impairment to these regions is thought to be responsible for the generation of migraine headache and vertigo. Thus, brain structure alterations in the vestibular and nociceptive pathways are important for exploring the pathogenesis of VM.

Voxel-based morphometry (VBM) is a common magnetic resonance imaging (MRI)-based technique that can be used to investigate structural abnormalities, including differences in brain gray matter (GM) volumes, between different populations (10). While several VBM studies of VM patients have been conducted, their results are inconsistent (11–14). For instance, one study found decreased GM volume in the temporal and occipital lobes (11), while another study suggested increased GM volume in the same brain regions (12). Given this variability, further analysis is needed to confirm reliable cerebral GM alterations in VM patients in order to inform potential pathogenesis and clinical treatments.

We hypothesized that VM patients exhibit spatially convergent GM alterations in brain regions critical for integrating vestibular and nociceptive signals, as suggested by their anatomical and functional connectivity. Identifying such alterations may provide a structural basis for the shared pathophysiology of vestibular and migraine symptoms in VM.

One approach to reconcile inconsistent results in imaging studies is coordinate based meta-analysis (CBMA) (15). Notably, this method has been previously applied to analyze voxel-based neuroimaging studies (16, 17). The latest version of CBMA is seed-based d mapping with a permutation of subject images (SDM-PSI) and shows increased accuracy relative to the former versions (18, 19).

This study aimed to explore whether VM patients show convergent GM alterations by conducting a CBMA via SDM-PSI of prior VBM studies comparing VM patient and healthy control (HC) groups. Overall, this work aims to shed light on the underlying neurophysiological mechanisms of this disease.

## Methods

### Study design

This study complies with the Preferred Reporting Items for Systematic Reviews and Meta-Analyses (PRISMA) checklist and follows the guidelines and recommendations of neuroimaging meta-analyses (20). It was pre-registered on the International Prospective Register of Systematic Reviews (PROSPERO, ID: CRD42021277684).

### Literature search

We searched the PubMed, Embase, and Web of Science databases using the following free-text terms: (“vestibular migraine”) AND (“MRI” OR “voxel-based” OR “morphometry” OR “morphometric” OR “structural” OR “cortical”). We also searched two Chinese databases (China National Knowledge Infrastructure and Wanfang) for studies published in Chinese. The reference lists of the included articles and relevant review articles were manually reviewed to identify other potentially eligible studies. We also searched bioRxiv^1^ for unpublished preprints eligible for inclusion. All searches were completed on 6 April 2024.

### Study selection

The inclusion criteria are as follows: (1) patients must meet the diagnostic criteria for VM in the International Classification of Headache Disorders 3rd version (ICHD-3); (2) studies must perform a VBM comparison between VM patients and healthy controls; (3) results are reported using Montreal Neurological Institute (MNI) or Talairach coordinates; and (4) studies are published in Chinese or English. The exclusion criteria are as follows: (1) articles that were not original research studies, such as reviews, case reports, reviews, editorial letters, conference reports, etc.; (2) studies without a healthy control group; (3) studies in which analysis was limited to defined regions of interest (ROIs); and (4) studies for which essential neuroimaging data were not available.

### Data extraction

Two researchers (DL and LH) independently reviewed the abstracts of the studied identified by the initial search to determine if they met the criteria. If a consensus could not be reached, a third expert was consulted. For all studies that met the inclusion and exclusion criteria, the following information was extracted: (1) publication information (first author’s name, title, journal, year of publication); (2) demographic information (number of participants per group, gender distribution, mean age); (3) clinical information [course of migraine, headache frequency, visual analog scale (VAS)]; (4) information about the MRI protocol (scanner manufacturer and platform, field strength, head coil, scan sequence, repetition time (TR), echo time (TE), voxel size); (5) information about data processing (imaging processing software package, smooth kernel, covariates, statistical threshold); and (6) main results (brain region, voxels, peak coordinates (x, y, and z), and corresponding t-statistics). Note that SDM-PSI can transform different statistical values; in this study, results are standardized as *t*-values.

### Quality assessment

The quality of each included study was assessed using a 10-point checklist based on a previous neuroimaging CBMA (21). This checklist primarily assesses the quality of the participants, methods, results, and conclusions (for further details, see Supplementary Table S1).

### Meta-analysis

Meta-analysis was performed using SDM-PSI software 6.21.^2^ SDM is a voxel-based meta-analysis method that allows researchers to integrate neuroimaging studies reporting peak coordinates (18, 19). This method has been previously applied to study a variety of neurological diseases.

The peak coordinates of significant GM differences in studies comparing VM patients and HCs were collected and organized. The standard SDM-PSI pipeline was then followed. Firstly, the maps of lower and upper bounds of possible effect sizes within a GM mask were calculated for each study after peak coordinates and effect sizes are entered into software. The effect sizes were then analyzed using MetaNSUE based on multiple imputations algorithms. Rubin’s rules were applied to integrate results across multiple imputations, combining within-imputation precision and between-imputation variance to generate pooled effect size estimates (18, 22). Finally, we used the default recommended preprocessing parameters [full width at half maximum (FWHM) = 20 mm, mask = gray matter] and performed 1,000 permutations. Voxel-wise results were determined using a threshold-free cluster enhancement family-wise error rate (TFCE-FWER) of *p* < 0.05 corrected for multiple comparisons and an extent threshold ≥10 voxels.

### Sensitivity analysis

Sensitivity analyses were conducted to confirm the stability of the CBMA results by excluding one study each time and repeating the analysis.

### Heterogeneity and potential publication bias analysis

Analysis of heterogeneity between studies was assessed using the I^2^ statistic; I^2^ > 50% was considered to reflect significant heterogeneity. The risk of potential publication bias was assessed by extracting significant values for the main CBMA peak using Egger’s test (23) with a threshold of *p* < 0.05.

### Meta-regression analysis

Meta-regression analyses were planned to investigate the potential effects of clinical variables (e.g., headache frequency, disease duration) on GM alterations. These analyses would have been conducted using the SDM-PSI framework, with statistical significance assessed at *p* < 0.05 (TFCE-based FWER correction) and a cluster extent threshold of ≥10 voxels.

### Interpretation with coordinated based databases

To further elucidate the cognitive functions associated with the brain regions identified in our meta-analysis (the coordinates [−44, −12, 16] and [48, −12, 8]), we conducted a post-hoc systematic functional decoding analysis using the Neurosynth and BrainMap databases. It was performed to provide a more comprehensive interpretation of our findings by linking the observed activation patterns to established neurocognitive domains. Specifically, the coordinates were queried in both databases to retrieve statistically significant associations with cognitive terms and activation profiles derived from a large corpus of neuroimaging studies. This approach, which has been validated in previous research (24), allowed us to contextualize the identified regions within a broader framework of cognitive function, thereby enhancing the interpretability and translational potential of our results.

## Results

### Study selection and characteristics

**Figure 1:**
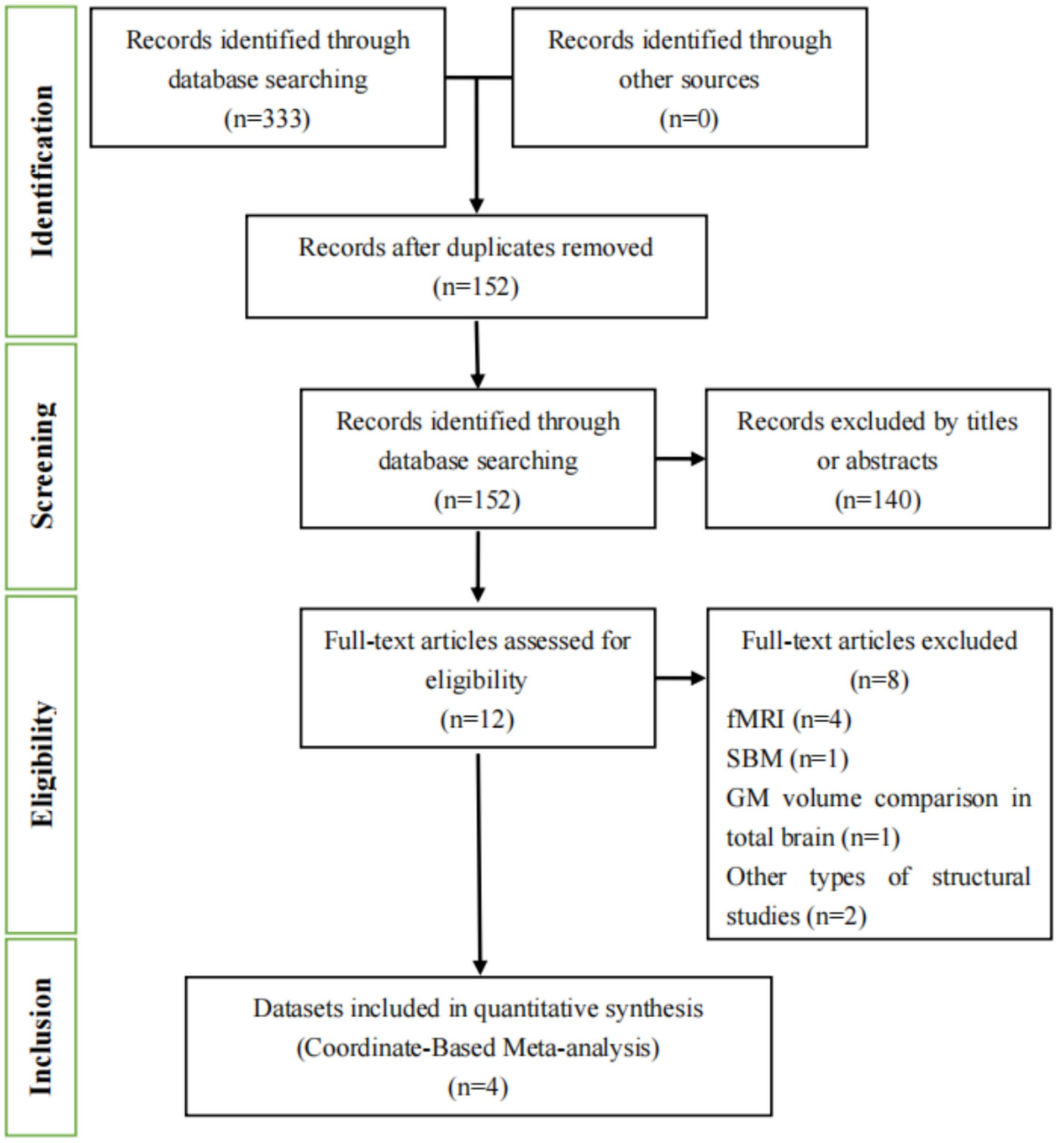
Flowchart of the literature selection process for the meta-analysis in English databases. fMRI, functional magnetic resonance imaging; SBM, surface-based morphometry; GM, gray matter.

**Figure 2:**
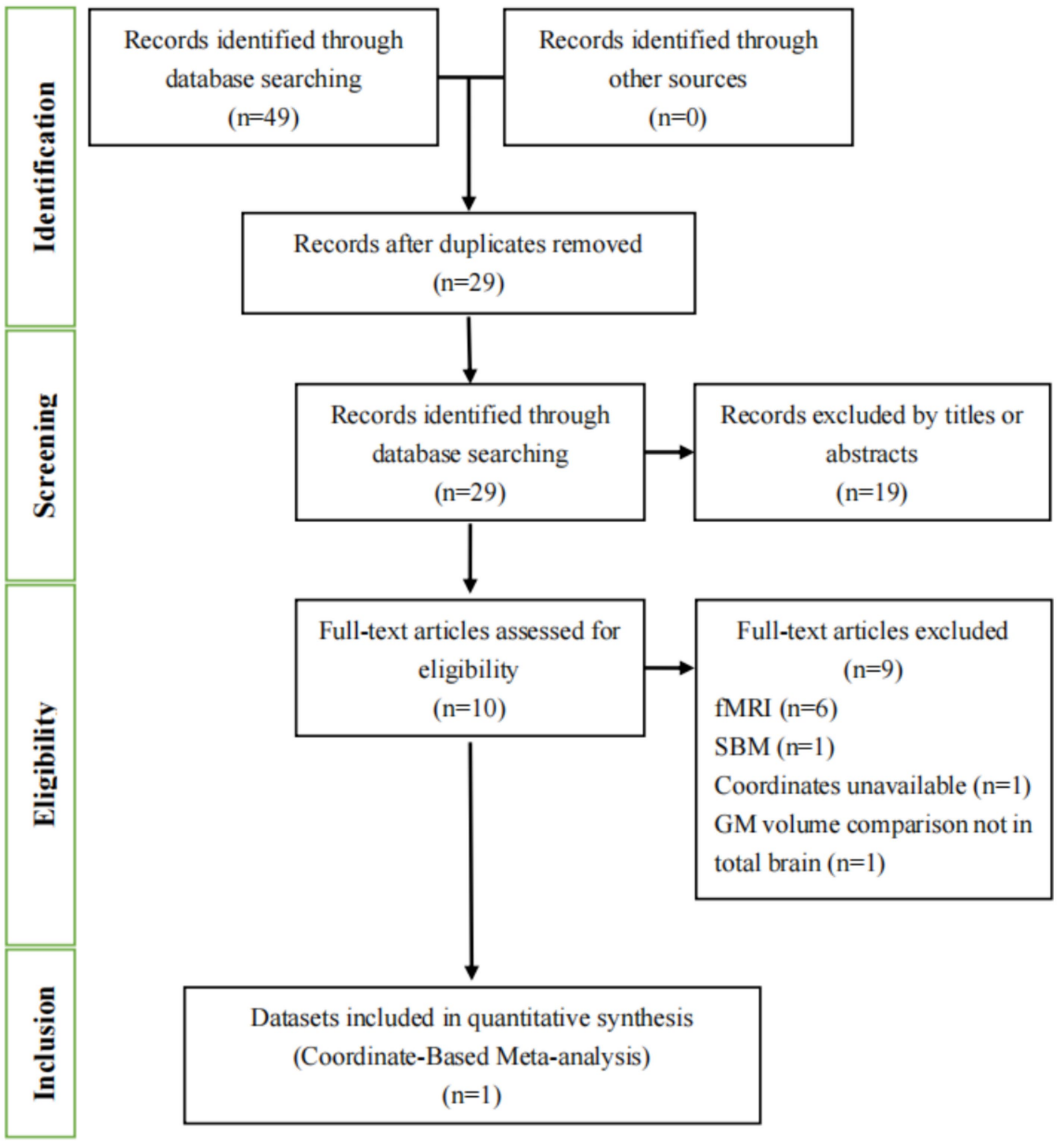
Flowchart of the literature selection process for the meta-analysis in Chinese databases. fMRI, functional magnetic resonance imaging; SBM, surface-based morphometry; GM, gray matter.

In our study, we conducted a comprehensive literature search using both English (Figure 1) and Chinese databases (Figure 2). In Figures 1, 2, we detail the process of literature screening and exclusion. In English databases, a total of 333 studies were screened, with 152 remaining after removing duplicates. Ultimately, 4 studies were included for CBMA analysis. The reasons for exclusion include: 140 studies excluded due to title and abstract not meeting research standards, 4 MRI-related studies, 1 surface-based morphometry (SBM) study, 1 whole-brain GMV comparison study, and 2 other types of structural studies. In Chinese databases, 49 articles were screened, with 29 remaining after removing duplicates. Ultimately, 1 study was included for CBMA analysis. The reasons for exclusion include: 19 studies excluded due to title and abstract not meeting research standards, 6 MRI-related studies, 1 SBM study, 1 study with unavailable coordinate data, and 1 study not involving whole-brain gray matter volume comparison.

Hence, five eligible studies were identified and their datasets were included in this study, which contained a total of 103 VM patients (83 female) and 107 HCs (82 female) (11–14, 25). Among the five included studies, four were identified from English databases and 1 from Chinese databases. The mean age did not differ significantly between the VM and HC groups [standardized mean difference = 0.23; 95% confidence interval (CI) = −0.04–0.50, z = 1.67, *p* = 0.094]. No significant difference in gender distribution was observed between VM patients and healthy controls (*χ*^2^ = 0.05, df = 1, *p* = 0.823), with comparable proportions of males [VM: 19.4% (20/103) vs. HC: 20.6% (22/107)] and females [VM: 80.6% (83/103) vs. HC: 79.4% (85/107)]. Of the five datasets, four reported headache frequency (mean frequency: 6.32 ± 4.04 days/month), four datasets reported VAS (mean score: 5.57 ± 2.06), and two reported DHI scores (mean scores: 47.83 ± 13.50). Paired comparisons confirmed no significant difference in sample sizes between VM and HC groups across studies [*t*(4) = −1.37, *p* = 0.241]. Additional details are provided in Tables 1, 2.

**Table 1:** Demographic and clinical characteristics of VBM studies were included in the meta-analysis.

Study	Sample (*n*)	Gender (Male /Female)	Age (Years ± SD)	Migraine history (Years ± SD)	Headache frequency (Days/month)	VAS (Mean, SD)
Obermann et al. (11)	VM = 17	3/14	42.71 ± 10.05	6.17 ± 4.51	6 ± 3.02	6.41 ± 1.88
	HC = 17	3/14	42.17 ± 9.26	–	–	–
Messina et al. (12)	VM = 19	7/12	40 ± 11.10*	15.7 ± 5.11*	6 (NA)	NA
	HC = 20	7/13	36.9 ± 9.6*	–	–	–
Zhe et al. (13)	VM = 20	2/18	38.60 ± 11.10	6.50 ± 6.27	6.90 ± 5.32	5.35 ± 1.84
	HC = 20	3/17	37.60 ± 11.84	–	–	–
Zhe et al. (14)	VM = 30	3/27	39.67 ± 11.10	8.39 ± 7.17	6.33 ± 4.89	5.23 ± 2.33
	HC = 30	4/26	37.67 ± 12.14	–	–	–
Wang et al. (25)	VM = 17	5/12	50.94 ± 12.13	5.62 ± 2.48	NA	5.60 ± 1.87
	HC = 20	5/12	45.50 ± 5.17	–	–	–

*The original text does not explicitly mention the standard deviation, based on the results of Furukawa et al. (60), the mean of the standard deviation of similar studies is taken instead.

**Table 2:** Imaging characteristics of the VBM studies were included in the meta-analysis.

Study	Scanner	Field	Head coil	Sequence	TR/TE (ms)	Voxel size (mm^3^)	Software	FWHM (mm)	Threshold	Quality score*
Obermann et al. (11)	Siemens Avanto	1.5 T	8 channel	MPRAGE	2400/3.53	1*1*1	SPM 8	10	*p* < 0.001 uncorrected	9
Messina et al. (12)	Philips	3.0 T	NA	3D-FFE	25/4.6	0.89*0.89*0.8	SPM 12	8	*p* < 0.05 (FWE) and *p* < 0.001 uncorrected	9
Zhe et al. (13)	Philips Ingenia	3.0 T	16 channel	MPRAGE	1900/2.26	1*1*1	SPM 12	8	*p* < 0.05 (FDR)	9
Zhe et al. (14)	Philips Ingenia	3.0 T	16 channel	MPRAGE	1900/2.26	1*1*1	SPM 12	8	*p* < 0.05 (FDR)	9
Wang et al. (25)	Philips	3.0 T	8 channel	3D-FFE	7.7/3.7	1*1*1	FSL	4	*p* < 0.001 (FWE)	8.5

### Main CBMA results

**Figure 3:**
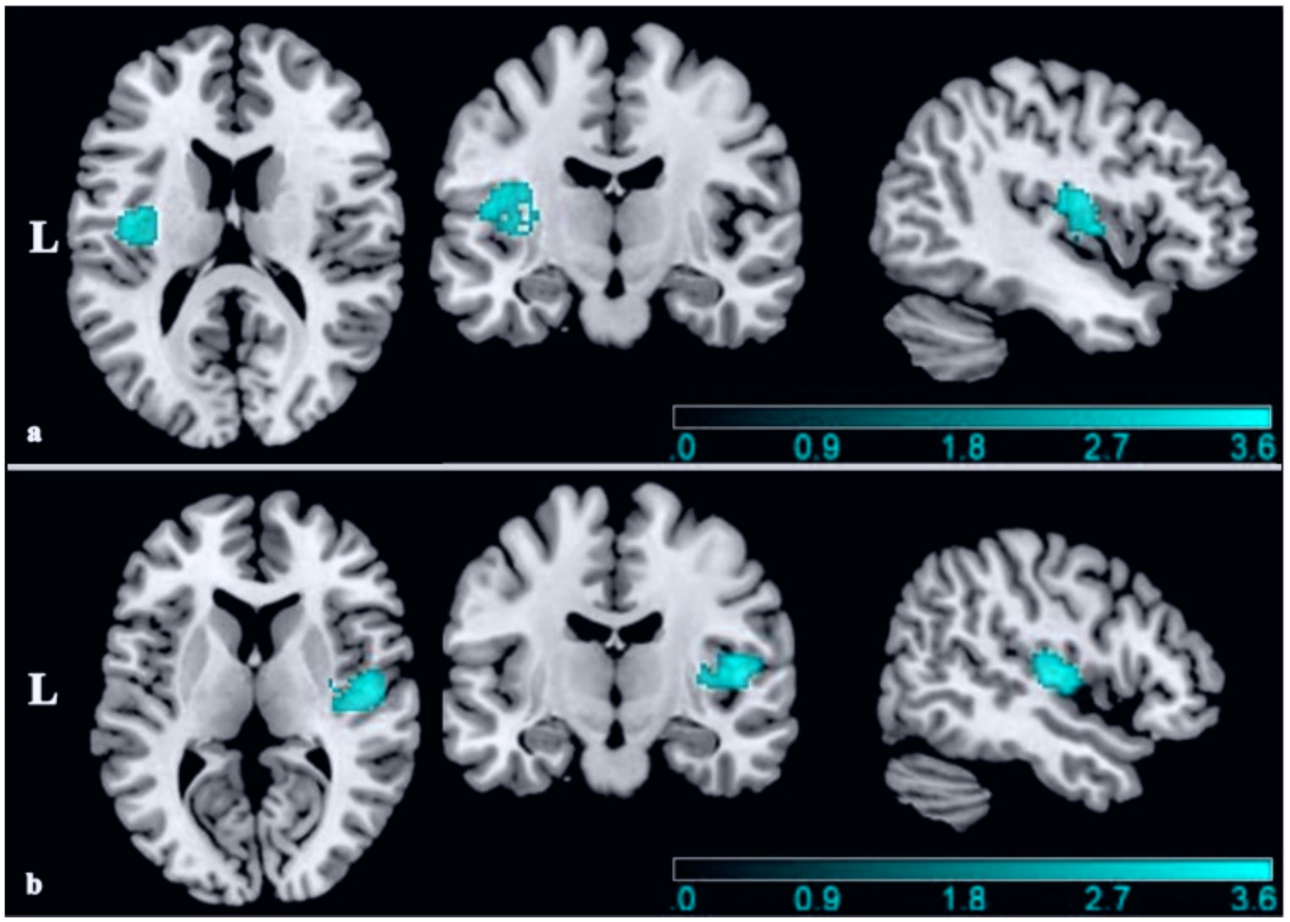
**(a)** The GM volume alterations in this meta-analysis when all datasets included (MNI coordinate: −44, −12, 16, SDM-Z = −3.68, TFCE corrected *p* = 0.004, Voxels = 629); **(b)** the GM volume alterations in this meta-analysis when Zhe 1 excluded (Exploratory finding after excluding a study with left-sided headache predominance) (MNI coordinate: 48, −12, 8, SDM-Z = −3.83, TFCE corrected *p* = 0.003 Voxels = 504). The bule color bar indicates the Z-value of the decreased GM volume. L, left; MNI, Montreal Neurological Institute; SDM, seed-based d mapping; TFCE, threshold-free cluster enhancement.

The CBMA results revealed a significant decreased GM volume in patients with VM compared with HCs in the left rolandic operculum (SDM-Z = −3.68 *p* = 0.004, voxels = 629) with the peak MNI coordinate [−44, −12, 16] located in Brodmann area (BA) 48. The two largest clusters belong to the insula (voxels = 126) and rolandic operculum (voxels = 173) (Figure 3a and Table 3).

**Table 3:** Regional differences in GM volume between patients with VM and healthy controls in the meta-analysis.

Condition	Brain regions	Peak MNI coordinate	SDM-Z value	TFCE *p*-value	Numbers of Voxels	Cluster breakdown
All datasets included	Left rolandic operculum	−44, −12, 16	−3.68	0.004	629	Left insula, BA 48 Left rolandic operculum, BA 48 Left lenticular nucleus, putamen, BA 48 Left Heschel gyrus, BA 48 Left superior temporal gyrus, BA 48 Left superior longitudinal fasciculus III Left fronto-insular tract 4 and 5 Left striatum
When Zhe 1 excluded	Right Heschel gyrus	48, −12, 8	−3.83	0.003	504	Right insula, BA 48 Right rolandic operculum, BA 48 Right heschl gyrus, BA 48 Right superior temporal gyrus, BA 48 Right lenticular nucleus, putamen, BA 48 Right fronto-insular tract 5 Corpus callosum

MNI, Montreal Neurological Institute; SDM, seed-based d mapping; TFCE, threshold-free cluster enhancement; BA, Brodmann area.

### Sensitivity analysis

We performed a total of five analyses. Even using TFCE-based FWER correction (*p* < 0.05), the left rolandic operculum region was present in 3 of 5 results. During these analyses, we incidentally observed that excluding Zhe et al. (13)—where 14/20 patients had left-sided headaches—resulted in GM reductions in the right Heschel’s gyrus (SDM-Z = −3.83 *p* = 0.003, voxels = 504) with the peak MNI coordinate [48, −12, 8] located in BA 48 (Figure 3b and Table 3). It is worth noting that the two largest clusters also belong to the insula (voxels = 243) and rolandic operculum (voxels = 173).

### Heterogeneity analysis and publication bias analysis

The I^2^ value obtained in this study was 19.35%, suggesting low heterogeneity between studies. The funnel plot showed no significant asymmetry in the left rolandic operculum. This result was confirmed by the Egger’s test (*p* = 0.826) (Supplementary Figure S1).

### Meta-regression analysis

Planned meta-regression analyses to explore the effects of clinical variables (e.g., headache frequency, disease duration) were not performed. The limited number of included studies (*n* = 5) falls below the recommended threshold for reliable meta-regression, which typically requires data from at least 10 studies to ensure sufficient statistical power and avoid overfitting.

### Cognitive function of the coordinates

In an effort to delve deeper into the cognitive functions associated with the coordinates we found, a comprehensive meta-analysis was conducted, utilizing both Neurosynth and BrainMap databases for the exploration of the specific sets of brain coordinates ([−44, −12, 16], [48, −12, 8]).

**Figure 4:**
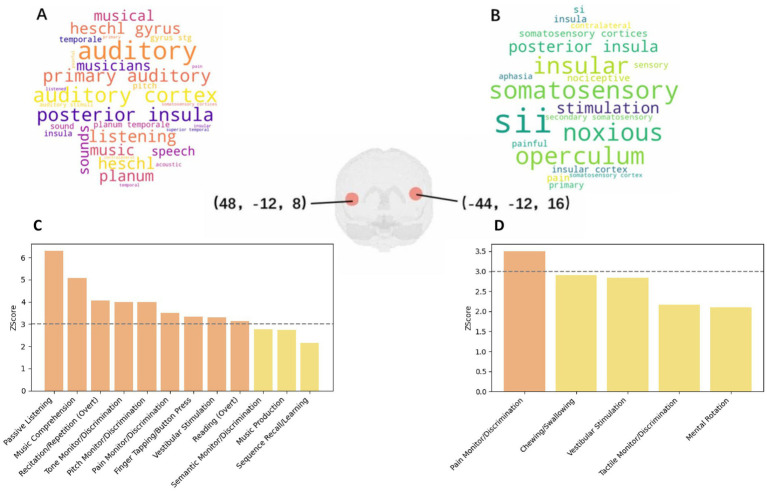
**(a)** Word Cloud for Coordinate [48, −12, 8] from Neurosynth; **(b)** Word Cloud for Coordinate [−44, −12, 16] from Neurosynth; **(c)** Paradigm Analysis for Coordinate [48, −12, 8] at nine radius; **(d)** Paradigm Analysis for Coordinate [−44, −12, 16] at nine radius.

Within the Neurosynth database, analysis of the coordinate [−44, −12, 16] resulted in the identification of 190 studies within a 6 mm radius. A manual review of these studies highlighted key articles related to the operculo-insular cortex, emphasizing interoceptive and multimodal functions pertaining to tactile, nociceptive, and vestibular representations (26), including studies on cold allodynia (27) and the functional connectivity of the vestibular cortex (28). Dominant cognitive terms associated with this coordinate were notably centered around “noxious” sensations and pain, as shown in Figure 4A.

Further insights were garnered through the Mango’s paradigm analysis, which did not reveal significant findings within a 6 mm radius but unfolded critical associations within larger radius: at 9 mm, associations with pain monitoring/discrimination were observed (Z-score = 3.504); expanding to a 12 mm radius further included vestibular stimulation (Z-score = 4.001), chewing/swallowing (Z-score = 3.869), and overt reading tasks (Z-score = 3.352), as shown in Figure 4C. Moreover, a notable association with schizophrenia was identified (Z-score = 3.79), alongside behavioral perceptions related to somesthetic pain (Z = 3.312).

The investigation of the coordinate [48, −12, 8] brought to light cognitive associations within a 6 mm radius, specifically related to music comprehension and passive listening, marking a stark contrast to the primarily pain-related findings of the previous coordinate. The related terms obtained from neurosynth are shown in Figure 4B. When the radius was increased, findings expanded to include pain monitoring/discrimination and vestibular stimulation, similar to the [−44, −12, 16] coordinate. Within broader radius, additional diseases such as schizophrenia (Z-score = 3.374), multiple sclerosis (Z-score = 3.242), and migraine (Z-score = 2.192, not significant) were correlated, underlining the multifaceted cognitive implications of these regions.

## Discussion

### Gray matter reductions in vestibular-migraine

In this study, we used an improved SDM-PSI method to quantify GM volume alterations in VM patients compared to HCs. This meta-analysis comprised 5 whole-brain VBM studies including 103 VM patients and 107 HCs. VM patients showed significantly decreased GM volumes in the BA 48 region, including the left insula and left rolandic operculum, compared to HCs. The left rolandic operculum GM reduction remained significant in 3/5 sensitivity analysis. Overall, these findings support that GM volume alterations do occur in VM patients, which may help us to further explore the pathophysiological mechanisms of VM.

In the five datasets included in this study, the finding of a reduction in GM volume in the left posterior insula–operculum region (left insula and left rolandic operculum) was present in three studies; similar brain regions were not reported in the other two studies (12, 25). Notably, Messina et al. (12) reported that VM patients had increased GM volume in frontal and occipital regions compared to HC, which was not found in other VBM studies. The following factors may have contributed to this result. Firstly, there are ethnic differences: the population in three of the five studies was Asian. Secondly, over half (11/19) of VM patients included in Messina et al.’s (12) study were taking anti-migraine medication. In addition, patients were required to be free of headache for 1 month prior to scanning, whereas other studies generally required 1–3 days free of headache before and after scanning, which may reflect GM volume alterations after treatment.

Previous VBM studies have shown that a wide range of brain regions are affected by VM, such as occipital, thalamic, and cerebellar regions (11–14, 25). In contrast, the GM volume alterations in this study were relatively concentrated in anatomically and spatially nearby brain regions. This difference may relate to the fact that some previous studies adopted more lenient statistical thresholds, such as not correcting for multiple comparisons (*p* < 0.001). The present study used a TFCE-based FWER correction (*p* < 0.05), which may have led to fewer positive findings. Different scanners, field strengths, sequences, TR/TE, data processing methods, and covariates in regressions may also have affected the results (29, 30). The clinical characteristics of the patients, such as the primary side of the headache attack, may also have affected the results. This is discussed in more detail below.

### Lateralization and pathophysiological implications

The observed GM alterations were predominantly localized to the left hemisphere, particularly in the rolandic operculum and posterior insula. This lateralization aligns with evidence of asymmetric vestibular processing, where the right hemisphere typically dominates vestibular function in right-handed individuals (31, 32). Notably, 4/5 studies explicitly recruited right-handed participants, and the left-lateralized findings persisted even when excluding Obermann et al. (11), which did not report handedness. While the incidental observation of right-lateralized GM reductions after excluding Zhe et al. (13) in sensitivity analysis suggested a potential association between headache laterality and GM asymmetry. The link to headache lateralization is exploratory, as laterality data were inconsistently reported. Obermann et al.’s (11) study did not mention the patients’ habitual hand, but the GM volume alterations remained localized to the left hemisphere when Obermann et al.’s (11) study was excluded in the sensitivity analysis. The relationship between headache laterality and structural changes remains unresolved. A prior study in migraine with aura found no GM differences tied to headache side (33), while others report contralateral cortical thinning in typical migraine. These discrepancies highlight the need for prospective studies with standardized laterality assessments. Our findings should not be overinterpreted to suggest a causal link between headache side and ipsilateral GM changes. Rather, they underscore the necessity of controlling for laterality in future VM imaging studies.

### Pathophysiological implications

The brain regions showing decreased GM volume in the present study were mainly part of the posterior insula and operculum regions, which both belong to the parieto-insular vestibular cortex (PIVC) (34). The PIVC is thought to be the core of the vestibular information processing center in macaque monkeys and is composed of the posterior insula, retroinsular region, and parietal operculum (35, 36); in humans, the equivalent brain regions might be the cytoarchitectonic area OP2 in the parietal operculum (28). Abnormalities in these regions have been reported in other types of neuroimaging studies of VM. A positron-emission tomography (PET) study reported increased metabolism in the posterior insula during the ictal phase compared to the interictal phase (37). A functional magnetic resonance imaging (fMRI) study revealed an altered blood oxygen level-dependent (BOLD) signal in this region (38). Our study supports these findings and suggests consistency between structural and functional alterations in these regions. Structural and functional alterations in PIVC regions can also be observed in other vestibular disorders, such as bilateral vestibulopathy (39) and chronic subjective dizziness (40). A VBM study of a stroke population also showed that structural damage in PIVC regions was closely associated with the development of vestibular symptoms (41). Therefore, it is reasonable to think that impairments to these brain regions cause vestibular symptoms.

More importantly, the posterior insula and operculum regions are also involved in processing of nociceptive information (42, 43). The posterior insula receives nociceptive afferents from the thalamic nucleus and interacts with brain regions such as the somatosensory cortex and cingulate gyrus, and the operculum is functionally analogous to the insula (9, 44–46). Structural and functional alterations in this region have also been reported in studies of episodic migraine (EM) and chronic migraine (CM) (47–51), which further confirm the findings of our study. Maleki et al. (49) showed decreased GM volume and functional activation in the insula in high-frequency migraineurs compared with low-frequency migraineurs, potentially implying that attack frequency leads to brain alterations. Schwedt et al. (52) constructed classifiers consisting of multiple structural MRIs that were highly accurate at identifying if patients had CM. Another study found that migraine duration may further increase the accuracy of classifiers (53). Taken together, these studies suggest that GM atrophy in the insula and its surrounding region correlate with the burden of migraine attack. Interestingly, vestibular syndrome often begins several years after typical migraine and is more common in CM (5). Given the heterogeneity of VM, we suggest that, for a subsample of VM patients, GM atrophy may be the result of recurrent headache attacks. The correlation between the headache side and GM atrophy side also suggests this possibility.

### Clinical applications

Regarding clinical applications, previous studies have shown that the regions around the insula may play an important role in migraine treatment (54–56). Triptans were shown to decrease activation in the posterior insular cortex compared with saline control (55). Moreover, it has been shown that the GM volume of the PIVC is negatively correlated with the Dizziness Handicap Inventory (DHI) score in VM patients (14). Considering the response of brain regions to anti-migraine treatments and its correlation with clinical indicators, it may serve as a target assessing the effects of treatment. Most importantly, we need to reduce headache attacks to reduce vertigo attacks.

Overall, the existing evidence is insufficient for causal deduction. It is not clear whether structural alteration in the PIVC region is the cause of vertigo or a compensatory response to impairment of the rest of the vestibular network. Although studies suggest GM atrophy to be hypofunctional (49, 57), this warrants further confirmation. The development of a multicenter imaging database of migraine patients and long-term follow-up studies will be important for elucidating these questions.

## Limitations

The primary limitations of this study stem from the scarcity of available neuroimaging data on vestibular migraine (VM), with only five studies meeting inclusion criteria. The small sample size (103 VM patients and 107 HCs across all datasets) precludes meta-regression analyses to evaluate the influence of clinical variables such as headache frequency, vertigo severity, or pain intensity, limiting our ability to disentangle migraine-specific contributions from vestibular-related GM alterations. Notably, all included studies had modest per-group sample sizes (<40 participants), which may amplify random error and reduce the stability of effect size estimates. Technical heterogeneity across studies—including variability in MRI protocols (1.5 T vs. 3.0 T scanners), statistical thresholds (uncorrected vs. FWE-corrected), and software pipelines (SPM vs. FSL)—further complicates the interpretation of spatially convergent findings, despite our use of TFCE-FWER correction to minimize false positives. While the SDM-PSI algorithm improves sensitivity over traditional CBMA methods, it relies on assumptions of unbiased reporting, potentially excluding studies with one-tailed statistical approaches. Additionally, post-hoc explorations of headache lateralization were limited by inconsistent reporting of symptom laterality in original studies and were not pre-specified in the PROSPERO protocol, necessitating cautious interpretation. Finally, the cross-sectional design precludes causal inferences regarding whether GM reductions in the posterior insula–operculum complex represent a predisposing neural vulnerability or a consequence of recurrent nociceptive-vestibular network activation, underscoring the need for longitudinal multimodal investigations (58, 59).

## Conclusion

CBMA confirmed a significantly reduced GM volume in VM patients. The GM volume alterations are mainly located in the posterior insula–operculum region, which is involved in both the nociceptive and vestibular networks system. Exploratory observations regarding headache lateralization warrant further investigation. Given that the causal relationship between structural alterations and the development of vestibular symptoms remains unclear, further structural and functional imaging paradigms, as well as longitudinal studies, are necessary to draw more precise conclusions. Furthermore, the headache side of the patient should be taken into account when designing migraine-related neuroimaging studies in the future.

## Data Availability

The original contributions presented in the study are included in the article/Supplementary material, further inquiries can be directed to the corresponding authors.

## Footnotes

^1^ https://www.biorxiv.org/

^2^ www.sdmproject.com
