# Supplementary material for: Coordinates-based meta-analysis for vestibular migraine and the underlying mechanisms behind it
Source: Front Neurol. 2025 Apr 9;16:1495423. doi: 10.3389/fneur.2025.1495423 (PMC12014765; doi:10.3389/fneur.2025.1495423)
Supplement: Supplementary file 1 [file Table_1.docx]

Table Supplement 1. Quality evaluation checklists

| **10-point checklist** |
| --- |
| **Category 1: Participants** |
| 1. Patients were evaluated prospectively, certain diagnostic criteria were used, and demographic data were reported. |
| 2. Healthy controls were evaluated prospectively; psychiatric and medical illnesses were excluded. |
| 3. Essential variables (e.g., age, gender, illness duration, symptom severity) were checked either via stratification or statistics. |
| 4. Both male and female participants were included and the sample size per group >10. |
| **Category 2: Methodology for image acquisition and process** |
| 5. Whole-brain level analysis was automated with no prior selection of regions. |
| 6. Spatial coordination was reported in standard space (e.g., Talairach or MNI* coordinates). |
| 7. The imaging technique utilized was clearly described for reproducibility. |
| 8. Measurements were clearly described for reproducibility. |
| **Category 3: Results and conclusions** |
| 9. Statistical parameters for both significant and critical no-significant differences were reported. |
| 10. Conclusions were consistent with the result and limitations were discussed |
|  |
| *Score* ***0 / 0.5 / 1*** *per item; total score out of 10; for criteria partially met, 0.5 points were given.* |
| **Total score** |
| MNI = Montreal Neurological Institute |


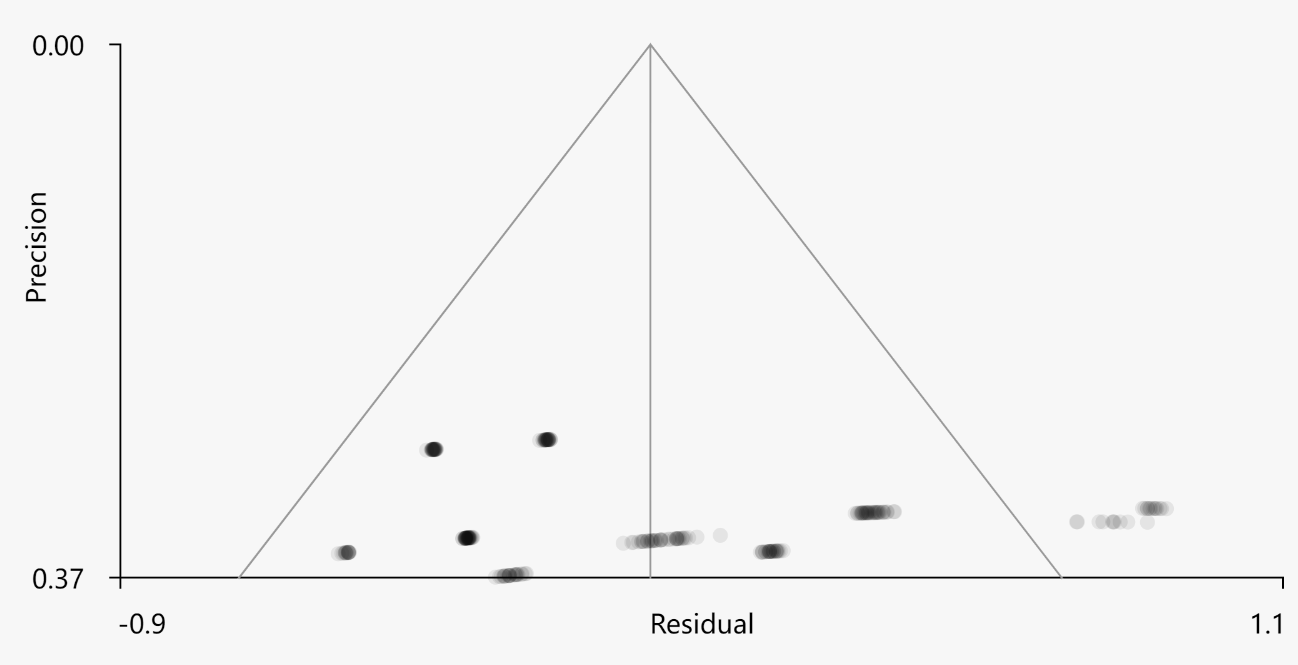


Figure Supplement 1. The funnel plot of this meta-analysis.
